# Incidence of Elbow Injury Patterns in Japanese Adolescent Judo Players: Analysis from a Nationwide Insurance Database

**DOI:** 10.3390/sports12110289

**Published:** 2024-10-23

**Authors:** Akira Ikumi, Eiji Sasaki, Naoki Sakuyama, Yasuo Mikami

**Affiliations:** 1 Department Orthopaedic Surgery, Institute of Medicine, University of Tsukuba, Tsukuba 3058575, Japan; 2 Department of Orthopedic Surgery, Hirosaki University Graduate School of Medicine, Hirosaki 0368562, Japan; e_sasaki@hirosaki-u.ac.jp; 3 Division of Frontier Surgery, The Institute of Medical Science, The University of Tokyo, Tokyo 1138655, Japan; saclibon@yahoo.co.jp; 4 Department of Rehabilitation Medicine, Kyoto Prefectural University of Medicine, Kyoto 6028566, Japan; mikami@koto.kpu-m.ac.jp

**Keywords:** elbow trauma, incidence, judo, nationwide database, sprain, dislocation, fracture-dislocation

## Abstract

Elbow injuries are common in judo, particularly among adolescents. This study investigated the incidence and patterns of three types of elbow injuries (sprain, dislocation, and fracture-dislocation) among Japanese adolescent judo players (12 to 18 years old) using data from a nationwide insurance database, covering the period from 2010 to 2019. A total of 4614 elbow injuries were recorded, with sprains being the most frequent (67.6% of cases). Female players had a 20% higher incidence of sprains than male players (6.8 vs. 5.4 per 1000 athlete-years). Severe injuries, such as dislocations and fracture-dislocations, were more common in high school players, with male middle school students exhibiting a higher rate of elbow fracture-dislocations (+15%) compared with female students. Elbow injuries frequently occurred during te-waza techniques, particularly seoi-nage, when players extended their arms to prevent being thrown. Peripheral nerve injuries, predominantly ulnar nerve injuries, accompanied elbow dislocations in 74% of the cases. This study highlights the importance of proper instruction in defensive and ukemi techniques, particularly those that discourage the use of hands to prevent falls during throws, to reduce the risk of elbow injuries.

## 1. Introduction

Judo is a Japanese martial art with approximately 20 million active participants across more than 200 countries globally [1]. Despite the International Judo Federation’s (IJF’s) efforts to prioritize athlete safety, the overall injury rate in judo competitions remains higher than that in many other sports [2]. Among these injuries, elbow injuries are particularly common, ranking third after knee and shoulder injuries according to Blach et al., who conducted a 16-year trauma survey in top-level European judo competitions [3]. In addition to severe head and neck injuries [4], serious elbow injuries requiring emergency transportation were also frequently observed during competition in our previous research [2].

**Figure 1:**
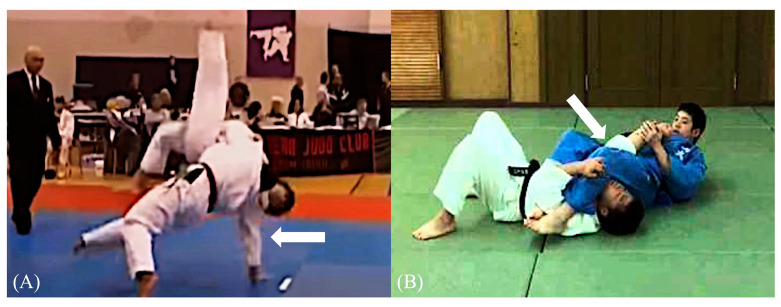
Injury situations of representative elbow injury in judo. (**A**) Thrown player puts hand out on tatami to defend, and left elbow is outstretched (white arrow), (**B**) right elbow is outstretched by joint-locking technique (white arrow). Source of figures: www.youtube.com/@JAPAN-JUDO, accessed on 17 October 2024).

**Figure 2:**
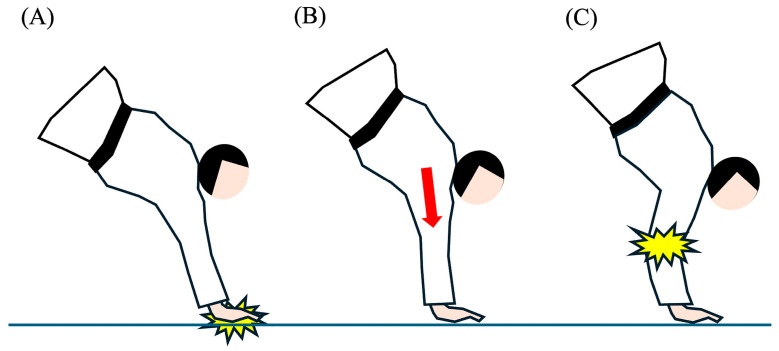
Wrong defense technique related to elbow injuries in judo. (**A**) Putting hand on tatami to avoid being thrown, (**B**) axial load (red arrow) applied to extended elbow, (**C**) outstretched elbow causes elbow injury.

Elbow injuries in judo occur under various scenarios, such as when competitors try to defend themselves by placing their hands on the mat (tatami) while being thrown, fall on their elbows, or experience joint-locking techniques (Figure 1). In judo, practitioners first learn ukemi, which are falling techniques intended to prevent injuries when being thrown or falling down. However, during randori (sparring practice) or competitions, players may try to protect themselves by putting their hands on the mat to avoid being thrown. In this situation, outstretching and axial compressive stress are applied to the elbow, which can often result in elbow injuries [5,6] (Figure 2). Although joint-locking techniques are permitted for competitors aged 15 and above, injuries can still occur if the player fails to submit in time or if the referee misses the submission. The biomechanics of elbow hyperextension and joint-locking techniques are critical in understanding the risks these actions pose, as the elbow joint can be exposed to extreme forces, leading to sprains (ligament injury), dislocations, or fracture-dislocations. Especially for middle and high school athletes who are in the process of physical development, the weakness of the epiphyseal plates makes them more prone to growth plate injuries during these scenarios [7].

Despite the prevalence of elbow injuries, comprehensive research on this issue, particularly among adolescent judo players, is limited. An epidemiological study of elbow dislocations in high school athletes indicated that elbow dislocations result in longer removal from play and are more likely to require surgical treatment than nondislocation-associated elbow injuries [8]. Although judo was not included in this study, wrestling exhibited a high injury rate compared to other sports, suggesting that judo, as a combat sport, also poses a high risk of elbow dislocation. Investigating the characteristics of elbow injuries among adolescent judo players who are in the process of physical development and have limited experience could be highly beneficial in formulating injury prevention programs and discussing rule changes.

Several Japanese researchers have recently used nationwide databases to examine the epidemiology of sports-related injuries [9,10,11,12]. By using nationwide databases, it becomes possible to comprehensively evaluate information on elbow injuries that occur across different competition levels and to generalize the findings. In this study, we utilized data from a nationwide insurance database to examine elbow injuries in adolescent judo players, with a particular emphasis on identifying mechanisms of injury and exploring potential preventive measures. The nationwide database provides a comprehensive overview, capturing data across various regions and competition levels, thus offering valuable insights into injury trends and prevention opportunities. The objectives of this study are to elucidate the factors contributing to elbow injuries and propose evidence-based preventive strategies to reduce injury incidence in young judo athletes.

## 2. Materials and Methods

### 2.1. Data Collection

This study analyzed elbow injuries in adolescents enrolled in middle or high school, based on data from the Injury and Accident Mutual Aid Benefit System, operated by the Japan Sports Council (JSC) [13]. The insurance system offers support to students from kindergarten to high school and provides payments when a medical consultation is completed, and an application is submitted to the JSC. Each application includes details such as the patient’s gender, date of birth, date of injury, activity at the time of injury, location, diagnosis, circumstances of the injury, treatment, and the sport involved. The hospital provides further details on the diagnosis, number of visits, and treatment costs. We analyzed data on three specific types of elbow injuries that occurred during physical education and athletic club activities from the submitted applications.

The following terms were searched to identify judo-related elbow injuries: judo, elbow injury, peripheral nerve injury related to elbow injury, injury situation, and throwing technique related to elbow injury. A total of 4614 elbow injuries were recorded over a 10-year period (January 2010–December 2019), categorized by school grade and age (7th–12th grades, ages 12–18).

In Japan, the majority of instructors and players are registered with the All-Japan Judo Federation (AJJF), as unregistered players are not allowed to compete, and instructors are prohibited from training them. Annually, approximately 200,000 individuals are registered in the AJJF. Between 2015 and 2019, 49,107–58,953 middle and high school judo players were registered each year. Over the 10-year period, the cumulative number of judo players included 80,854 male and 26,594 female 7th graders, with similar distributions across other grade levels.

### 2.2. Classification of Injuries

Elbow injuries were classified as sprains, dislocations, or fracture-dislocations, and minor injuries (contusions, bruises, lacerations) were excluded. Injuries were further classified based on situations (categorized standing offense, standing defense, fall down, ground, joint-locking, others, unknown) and related throwing techniques. In judo, there are 68 throwing techniques, categorized into five types: te-waza, koshi-waza, ashi-waza, ma-sutemi-waza, and yoko-sutemi-waza [14]. Te-waza consists of effectively using the hands/arms to throw the opponent, such as seoi-nage. Koshi-waza consists of throwing an opponent in a sudden motion, using one’s hip as the fulcrum, such as harai-goshi. Ashi-waza consists of using one’s foot/leg for reaping, tripping, sweeping, supporting, or entangling, such as uchi-mata. Sutemi-waza consists of Tori (player-executing technique) wrapping Uke’s (player receiving opponent’s attack) body around their own and falling together with them. In Ma-sutemi-waza, Tori sacrifices their own posture in the backward direction while throwing Uke over their own head, such as Tomoe-nage. In Yoko-sutemi-waza, Tori sacrifices their posture laterally, using that force to throw their opponent, such as Yoko-gake.

### 2.3. Incidence Calculation

The age- and sex-specific incidence of elbow injury was estimated as incidence per 1000 athlete-years, based on the number of elbow injuries recorded in the JSC database and the number of judo players registered with the AJJF [10]. Furthermore, the age- and sex-specific concomitant injury rates of peripheral nerve injury with elbow injury were estimated.

### 2.4. Statistical Analysis

Statistical analyses were conducted using SPSS software (version 27.0; SPSS Inc., Chicago, IL, USA). A significance level of *p* < 0.05 was set for all analyses. The distribution of elbow injuries between middle and high school, sexes, school grades, as well as the distribution of injury circumstances, was analyzed using the Chi-square test to determine differences in frequencies. In addition, for each type of injury, the distribution of injury circumstances and the incidence of peripheral nerve injuries were also analyzed using the Chi-square test. For items where a significant difference was observed (*p* < 0.05), adjusted residuals were used to identify which specific frequencies were significantly higher or lower. Frequencies with adjusted residuals greater than +1.96 or less than −1.96 were considered significantly different compared to other frequencies.

### 2.5. Ethical Considerations

This study was conducted in accordance with the Declaration of Helsinki and approved by the Institutional Review Board of Takahagi Kyodo Hospital (2024-8, 17 September 2024).

## 3. Results

### 3.1. Distribution of Elbow Injuries

Among the 4614 cases of judo-related elbow injuries, comprising 3120 sprains, 816 dislocations, and 678 fracture-dislocations, the distribution of elbow injuries between middle school and high school, sex, and school grade exhibited a significant difference in the distribution of elbow injuries (*p* < 0.001, *p* = 0.003, and *p* < 0.001). In the examination of the differences in frequencies using adjusted residuals (ARs), sprains were significantly more prevalent in middle school players (AR = 6.8) and female players (AR = 3.1), whereas dislocations were significantly more prevalent in high school players (AR = 8.4), and fracture-dislocations were significantly more prevalent in male players (AR = 3.0) (Table 1).

### 3.2. Incidence Rates of Elbow Injuries

**Figure 3:**
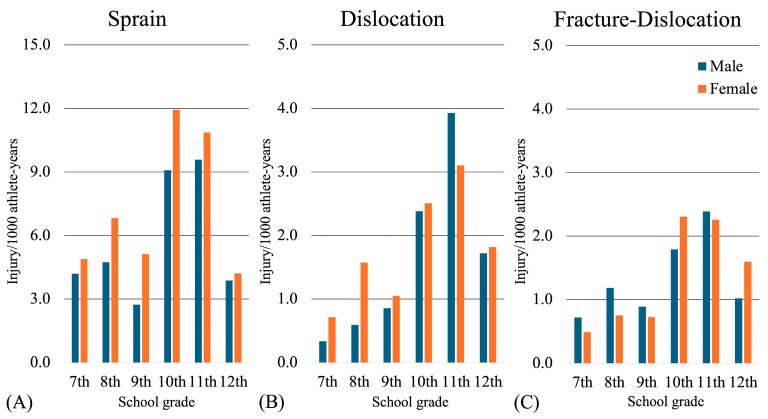
Age-specific incidence of elbow injuries in judo players (per 1000 athlete-years). (**A**) Sprain, (**B**) dislocation, (**C**) fracture-dislocation. The graphs show the incidence rates for male (dark bars) and female (light bars) judo players from 7th to 12th grade, highlighting variations in injury rates by age and sex.

The incidence rates of elbow injuries (per 1000 athlete-years) between the sexes (boys/girls) were as follows: 5.4/6.8 for elbow sprains, 1.5/1.6 for elbow dislocations, and 1.3/1.2 for elbow fracture-dislocations. Furthermore, the examination of the incidence rates of elbow joint injuries by grade revealed a peak in all injuries in grades 10 and 11. Elbow sprains exhibited a higher incidence rate among girls across all grade levels, whereas elbow dislocations displayed a higher incidence rate among boys only in the 11th grade. For elbow fracture-dislocations, boys showed a higher incidence rate than girls, not only in the 11th grade, but also across all middle school grades (Figure 3).

### 3.3. Elbow Injuries During Physical Education and Club Activities

**Figure 4:**
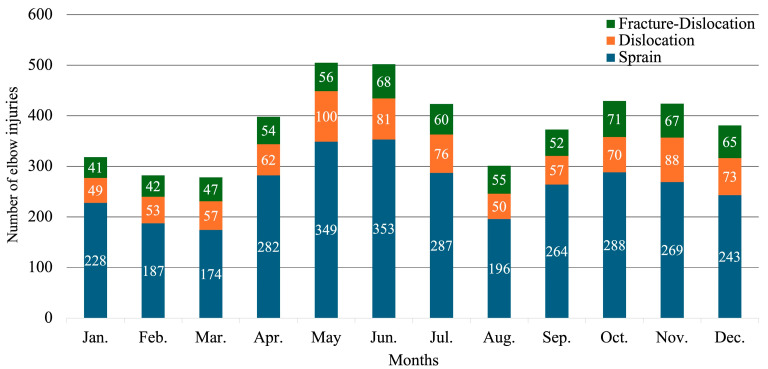
The number of monthly elbow injuries and their injury type. The number of occurrences shows a bimodal peak, which corresponds to the competition periods for middle and high school students in Japan.

The number of elbow injuries during physical education and club activities were as follows: In physical education, there were 539 elbow sprains, 166 elbow dislocations, and 109 elbow fracture-dislocations. In club activities, there were 2581 elbow sprains, 650 elbow dislocations, and 569 elbow fracture-dislocations. The occurrence ratios (club activities/physical education) were 4.8 for elbow sprains, 3.9 for elbow dislocations, and 5.2 for elbow fracture-dislocations, indicating a higher incidence of all injury types associated with club activities. The monthly occurrence counts for elbow injuries exhibited a bimodal pattern, with peaks in May–June and October–November, displaying a dual-peak trend. Elbow injuries were most frequent in May, with the risk being 1.8 times higher in March (Figure 4).

### 3.4. Injury Circumstance of Elbow Injuries

**Figure 5:**
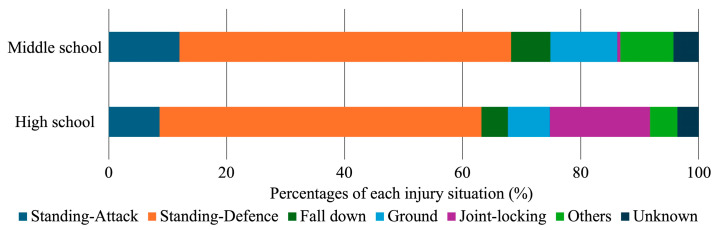
Distribution of injury situations in middle and high school judo athletes. The horizontal bars represent the percentage of injuries in different situations. The data show that both middle and high school athletes experience injuries across various judo situations, with noticeable proportions of injuries resulting from joint-locking techniques, particularly in high school athletes.

The most frequent circumstance of elbow injury occurred when a player was thrown by an opponent and put their hand out on the tatami, in both middle (56.2%) and high (54.6%) school players. Joint-locking techniques are permitted in high school, comprising 17.0% of all elbow injuries. There was a significant difference in injury circumstances between middle and high school students (*p* < 0.001). Using adjusted residuals, elbow injuries due to attacks while standing, falling from a standing position, and ne-waza showed significantly higher percentages in middle school students (adjusted residuals = 3.7, 3.3, and 5.0, respectively), while elbow injuries due to joint-locking techniques showed significantly higher percentages in high school students (adjusted residual = 18.3) (Figure 5).

**Figure 6:**
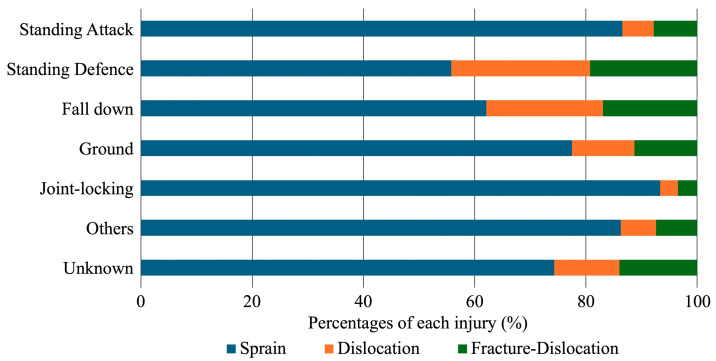
Detailed distribution of injury situations for each type of elbow injury. The horizontal bars represent the percentage of different elbow injuries across various judo situations. The data highlight the varied occurrence of specific injuries depending on the judo situations involved, with sprains being the most frequent injury type across all situations. Additionally, the percentages of dislocations and fracture-dislocation significantly increase in standing-defense and fall-down situations, while they significantly decrease in joint-locking situations.

The injury circumstances for each injury type showed significant differences (*p* < 0.001). Upon examining the differences in frequencies using adjusted residuals, elbow sprains significantly increased in the standing attack, ne-waza, and joint-locking techniques (adjusted residuals = 9.2, 4.5, and 12.5), while elbow dislocations and fracture-dislocations significantly increased in the standing defense (being thrown by an opponent) (adjusted residual = 14.4 for elbow dislocations and 9.7 for elbow fracture-dislocations) (Figure 6).

### 3.5. Throwing Techniques Related to Elbow Injuries

**Figure 7:**
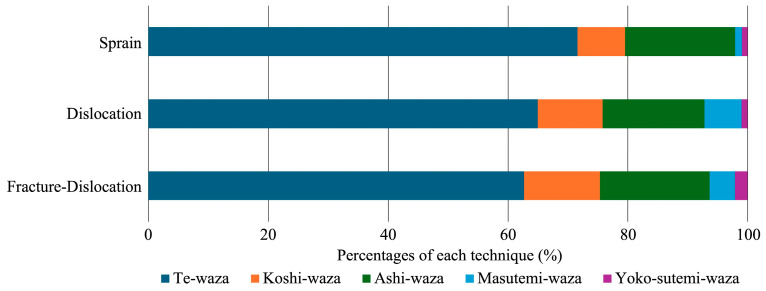
Technique-related elbow injuries expressed in percentages for all three elbow injuries (sprain, dislocation, and fracture-dislocation).

The most common elbow-injury-related throwing techniques were te-waza, such as seoi-nage and tai-otoshi, followed by ashi-waza, such as osoto-gari and uchi-mata, and koshi-waza, such as harai-goshi and sode-tsurikomi-goshi. The proportion of injuries resulting from sutemi-waza, such as tomoe-nage, increased in elbow dislocations, and fracture dislocations (Figure 7).

### 3.6. Peripheral Nerve Injuries Associated with Elbow Injuries

There were 23 cases of peripheral nerve injury associated with elbow injuries in this study, with ulnar nerve injuries comprising the majority of cases (n = 17, 74.0%). There were significant differences between the elbow injury types and the occurrence of peripheral nerve injuries (*p* = 0.002). Examining the differences in frequencies using adjusted residuals revealed a significantly higher occurrence of nerve damage associated with elbow dislocations (adjusted residual = 2.7), while this was significantly lower in cases of sprains (adjusted residual = −3.4). No significant associations were found between middle and high school age, sex, injury circumstances, or throwing techniques with regard to elbow injuries.

## 4. Discussion

### 4.1. Prevalence and Trends of Elbow Injuries in Judo

Sprains were the most common elbow injuries related to judo among Japanese adolescents, with a significant increase in dislocations occurring during high school, with peaks in the 11th grade. Although the overall incidence rate was higher among girls, elbow dislocations were more frequent among boys in high school, while elbow fracture-dislocations were more common among boys in middle school. Injuries occurred more often during club activities than during physical education, showing a bimodal peak in May–June and October–November. Over half of the injuries occurred when defending a throwing technique, and nearly 20% of the injuries in high school were caused by joint-locking techniques that are permitted at this level.

### 4.2. Risk Factors of Elbow Injuries in Judo

Sports injuries and disabilities result from a combination of various risk factors, broadly categorized as internal and external risk factors [15]. The internal risk factors include the strength, flexibility, and skill level of each athlete. Additionally, physical growth and development according to age impact the occurrence of sports injuries during adolescence. This study found that the overall incidence of elbow injury was higher in girls. Several female hormones influence joint laxity through their effects on collagen metabolism, ligament remodeling, and structural integrity [16,17,18]. Relaxin, a hormone that peaks during the luteal phase of the menstrual cycle, binds strongly to the tissues supporting joints [17]. Estrogen and progesterone both play crucial roles in ligamentous laxity, with estrogen decreasing collagen synthesis while progesterone exerts the opposite effect, contributing to variations throughout the menstrual cycle [18]. These hormones can alter collagen and collagenase production, thereby affecting the mechanical properties of the ligaments and potentially increasing joint laxity and injury risk. Several observational studies have associated a peak in relaxin levels with an increased risk of anterior crucial ligament injury, which is an additive risk per menstrual cycle [19,20,21]. Although no studies explicitly stated that female hormones increase the risk of elbow injuries in sports, the findings of this study suggest that the joint laxity influenced by female hormone levels [16] may potentially increase the risk of elbow injuries, similar to the risk observed in knee injuries. In addition, as women generally possess weaker muscle strength especially in upper body than men [22], the risk of elbow injuries may become higher when the hands are placed on the tatami to defend from opponent’s throwing. Therefore, learning the correct ukemi techniques and instructing adolescent athletes not to use their hands when being thrown are effective methods to avoid injuries.

In contrast, this study found that the incidence rate of elbow fracture-dislocations was higher among boys in middle school. Generally, closure of the growth plate occurs later in boys than in girls; epiphyseal closure of the proximal radius and ulna is complete at the age of 16 in boys and 14 in girls [23]. The epiphyseal plate is a cartilaginous region between the epiphysis and metaphysis, which is crucial for bone growth, but is also a point of vulnerability during periods of rapid growth. This vulnerability is due to the relative weakness of the plate compared with the surrounding muscle and tendon structures, increasing its susceptibility to fractures from shearing, splitting, or compressive forces [24]. The findings of this study suggest that the frequency of elbow fracture-dislocations was higher in middle school boys before the closure of the growth plate compared to girls, possibly due to sex differences in bone growth. Therefore, special attention should be paid to the possibility of fractures accompanying elbow dislocation in middle school boys.

External risk factors in the judo environment include the size of the dojo, density of participants (space per athlete), quality of the tatami (material and hardness), and the presence of springs and protective mats on the walls and pillars. Although this study did not identify many injuries caused by environmental factors, creating a safe and competitive environment is important for injury prevention. Ensuring sufficient distance from other participants and obstacles during partner practice, such as throwing and randori, is crucial for preventing injuries. In addition, extrinsic factors, such as coaching and training, are crucial for injury prevention. In adolescent sports activities, practice schedules are often determined by coaches, and the content and intensity of practice may affect the occurrence of injuries. We found that the peak periods for elbow injuries were from May to June and October to November. These periods coincide with the competition seasons for middle and high school students in Japan. During these periods, the proportion of randori in preparation for competitions is expected to increase. Since this study found that most elbow injuries in judo occurred during randori and competition, it is important to consider the increased risk of elbow injuries during these periods. Emphasizing the risk of elbow injuries and thoroughly instructing athletes on proper ukemi techniques, including not using their hands when being thrown, is crucial.

### 4.3. Mechanism of Elbow Injuries in Judo

**Figure 8:**
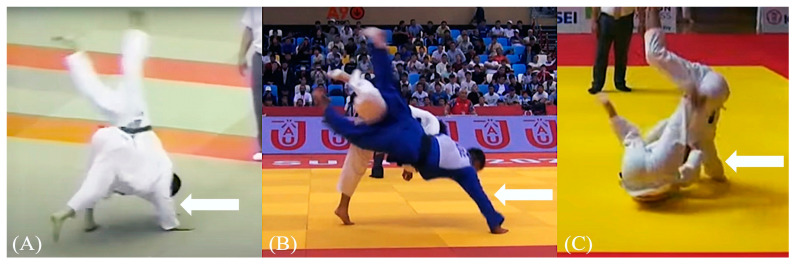
Typical injury situation by throwing technique. (**A**) Seoi-nage, (**B**) uchi-mata, (**C**) tomoe-nage. Player being thrown puts their hand on tatami to prevent being thrown (white arrow). Source of figures: www.youtube.com/@JAPAN-JUDO, accessed on 17 October 2024.

The results of this study revealed that the characteristics of throwing techniques also affected the occurrence of elbow injuries. Among the three types of elbow injuries, 60%–70% occurred during the te-waza technique, followed by the ashi-waza technique. Additionally, the proportion of dislocations and fracture-dislocations was higher during the masutemi-waza technique than sprains. Considering the characteristics and injury mechanisms of each technique, we found that the te-waza technique, especially in seoi-nage, was a common cause of injury. Seoi-nage involves ducking under the opponent’s arm and lifting and throwing them forward. This technique often leads to scenarios in which the thrown player extends a hand to defend against being thrown, resulting in elbow outstretching and subsequent injury. Uchi-mata, an ashi-waza technique, involves sweeping the opponent’s leg forward. Similar to seoi-nage, the extended hand used to defend against being thrown leads to elbow injuries. Tomoe-nage, a Masutemi-waza technique, involves falling backward and kicking the opponent over the head to throw them. Injuries occur when the thrown player extends a hand to the tatami, causing elbow outstretching. Techniques such as ura-nage, which involves backward throws, also lead to elbow injuries when players extend their hands when being thrown (Figure 8). Therefore, mastering the correct ukemi that avoids hand use is crucial for preventing elbow injuries. In situations in which hand use to protect the head, face, or spine is unavoidable, avoiding elbow extension is also important to prevent elbow injuries.

### 4.4. Peripheral Nerve Injuries Associated with Elbow Injuries in Judo

This study found 23 cases of nerve injuries associated with elbow injuries. The major peripheral nerves of the upper limb, including the ulnar, median, and radial nerves, are transverse to the elbow joint. Known nerve injuries related to fractures around the elbow include posterior interosseous nerve injuries associated with Monteggia fracture-dislocations and median nerve injuries associated with supracondylar humeral fractures [25,26]. This study found that a high frequency of ulnar nerve injuries was associated with elbow dislocation. The ulnar nerve passes through the medial side of the humerus and behind the medial epicondyle through the cubital tunnel to the forearm. Elbow dislocations in judo often occur when a player extends the hand during a fall, leading to significant displacement and excessive traction or compression of the ulnar nerve, causing injury. Nerve injuries can result in irreversible damage, emphasizing the importance of the prevention of elbow dislocations that may cause nerve injuries. Proper ukemi techniques to avoid hand use during falls are essential for this prevention.

### 4.5. Limitations

This study has several limitations. Firstly, there is the selection bias caused by focusing solely on students aged 12 to 18. Further research is necessary to evaluate the trends in other age groups. Secondly, this study did not account for biological age. Biological age, especially peak height velocity, is well known as a vulnerable phase for injury occurrence in youth. Accordingly, evaluating injuries by biological age is necessary in the future. Thirdly, this study relied on registry data, so we were unable to verify the accuracy of the diagnoses or treatment details. Information about elbow injuries was drawn only from the descriptions in the application documents, which were often incomplete. Finally, biomechanical analysis to explore the injury mechanisms was not feasible. Important risk factors for elbow injury in judo, such as detailed body measurements, body composition, weight classes, performance levels, and environmental conditions, were not examined in this study.

## 5. Conclusions

Elbow injuries are more prevalent among adolescent judo players, particularly girls. Sprains are the most common type of injury, whereas severe elbow injuries such as dislocations and fracture-dislocations occur more frequently in high school. These injuries commonly occur when players use their hands to defend against being thrown, particularly in te-waza techniques. Preventive strategies should focus on instructing athletes in proper defensive and ukemi techniques to avoid hand use when being thrown and reduce the risk of elbow injuries. Coaches should also consider adjusting training intensity during competitive periods to prevent elbow injuries.

## Figures and Tables

**Table 1:** The distribution of elbow injuries. * Chi-square test.

	Sprain	Dislocation	Fracture-Dislocation	Total	*p* Value *
Category					
Middle school	1405	233	283	1921	*p* < 0.001
High school	1715	583	395	2693	
Sex					
Male	2293	623	539	3455	*p* = 0.003
Female	827	193	139	1159	
Gender					
7th	469	46	71	586	
8th	582	92	120	794	
9th	354	97	92	543	*p* < 0.001
10th	698	174	137	1009	
11th	727	279	175	1181	
12th	290	128	83	501	
Total	3120	816	678	4614	

## Data Availability

The study protocol, statistical analysis, and data supporting the findings of this study are available from the corresponding author upon reasonable request.
